# The TOR–Auxin Connection Upstream of Root Hair Growth

**DOI:** 10.3390/plants10010150

**Published:** 2021-01-13

**Authors:** Katarzyna Retzer, Wolfram Weckwerth

**Affiliations:** 1 Laboratory of Hormonal Regulations in Plants, Institute of Experimental Botany, Czech Academy of Sciences, 165 02 Prague, Czech Republic; 2 Molecular Systems Biology (MOSYS), Department of Functional and Evolutionary Ecology, University of Vienna, 1010 Vienna, Austria; wolfram.weckwerth@univie.ac.at; 3 Vienna Metabolomics Center (VIME), University of Vienna, 1010 Vienna, Austria

**Keywords:** TOR signaling, auxin, PIN-FORMED 2, root hair growth, polar cell elongation, ROP2, ROS, root growth, plant adaptation

## Abstract

Plant growth and productivity are orchestrated by a network of signaling cascades involved in balancing responses to perceived environmental changes with resource availability. Vascular plants are divided into the shoot, an aboveground organ where sugar is synthesized, and the underground located root. Continuous growth requires the generation of energy in the form of carbohydrates in the leaves upon photosynthesis and uptake of nutrients and water through root hairs. Root hair outgrowth depends on the overall condition of the plant and its energy level must be high enough to maintain root growth. TARGET OF RAPAMYCIN (TOR)-mediated signaling cascades serve as a hub to evaluate which resources are needed to respond to external stimuli and which are available to maintain proper plant adaptation. Root hair growth further requires appropriate distribution of the phytohormone auxin, which primes root hair cell fate and triggers root hair elongation. Auxin is transported in an active, directed manner by a plasma membrane located carrier. The auxin efflux carrier PIN-FORMED 2 is necessary to transport auxin to root hair cells, followed by subcellular rearrangements involved in root hair outgrowth. This review presents an overview of events upstream and downstream of PIN2 action, which are involved in root hair growth control.

## 1. Introduction

**Figure 1:**
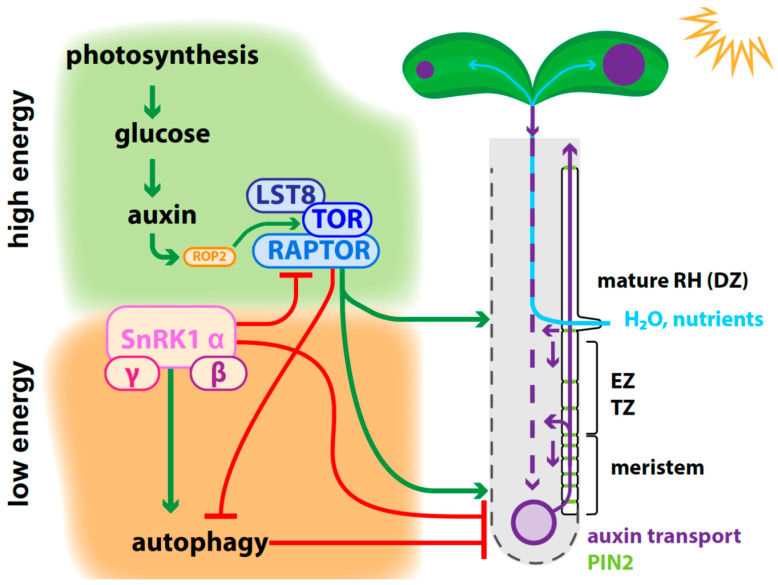
Energy status and resource uptake are orchestrated by an interplay of TARGET OF RAPAMYCIN (TOR) and SNF1-RELATED KINASE 1 (SnRK1) complexes, which antagonistically modulate root and root hair growth. High energy status results from sugar synthesis derived from photosynthesis in the leaves, which is followed by elevated auxin biosynthesis. Auxin signaling enhances RHO-RELATED PROTEIN FROM PLANTS 2 (ROP2) activity, and ROP2 binds TOR and promotes its phosphorylation. This results in the activation of the TOR complex, which further consists of the substrate-presenting subunit REGULATORY-ASSOCIATED PROTEIN OF TOR (RAPTOR) and the complex stabilizer LETHAL WITH SEC THIRTEEN 8 (LST8), described in Section 2.1. TOR signaling positively correlates with root meristem activity and therefore with root length. TOR stabilizes the auxin efflux carrier PIN2 by phosphorylation at the plasma membrane, and thereby modulates shootward auxin transport. Auxin levels in the meristem enhance the cell proliferation rate and are involved in root hair cell priming (described in Section 3.1). Furthermore, efficient auxin delivery and signaling trigger root hair elongation, which is responsible for root surface enlargement, allowing enhanced uptake of water and nutrients to ensure plant growth and productivity. Under high energy levels, TOR signaling inhibits autophagy, which would trigger the engulfment and recycling of existing plant material to gain resources, and this is accompanied by the end of root growth. When the energy levels in the root drop, reduced sugar availability is sensed over the SNF1-RELATED KINASE 1 (SnRK1) complex, which balances between TOR complex deactivation and autophagy activation. SnRK1 complex activity results in RAPTOR deactivation by phosphorylation, and the downregulation of auxin efflux transporters involved in auxin transport from the shoot to the root, followed by reduced root meristem activity (described in Section 3.2). EZ = elongation zone; TZ = transition zone; DZ = differentiation zone.

Plant growth, and, more critically, its adaptation to ever-changing environmental conditions, is maintained by the ability of plants to perceive exogenous signals, to evaluate the availability of resources, and to initiate signaling cascades orchestrating changes in growth rate and direction [1,2,3]. The most crucial aspect of plant fitness is to keep the potential of balancing the amount of cell division in meristematic regions and elongation processes and differentiation to mature cells, which depends on environmental conditions and available resources [2,4]. Efficient growth requires the generation of energy in form of sugars in the leaves of the plant upon photosynthesis, which is followed by the biosynthesis of the phytohormone and growth regulator auxin [2,5,6]. Furthermore, the plant needs to regulate nutrient and water uptake through the root from the soil [7,8]. To enhance uptake efficiency, the root has the ability to grow tubular structures from specialized epidermis cells, the root hairs [7,8]. Root hair cell priming and outgrowth are highly dependent on proper auxin distribution and abundance, whereby both are dependent on environmental conditions and the overall condition of the plant [2,8,9,10,11]. Distribution of the phytohormone auxin through the plant creates auxin gradients, which orchestrate the activity of meristems, adaptational growth via cell expansion, and differentiation of specialized cells [12,13,14,15]. PIN-FORMED (PIN) proteins, auxin efflux carriers, ensure on-point and polar distribution of the phytohormone, whereby auxin signaling underpins the fine-tuning of various cellular processes involved in plant growth adaptation under changing environmental conditions [3,16,17,18]. The role of the root-specific PIN family member PIN2 during root hair growth adaptation in response to environmental conditions will be discussed in particular in the scope of this review. The establishment of auxin gradients is crucial for plant growth from the first asymmetric cell division of the zygote [19,20] and tissue organization [21] through fine-tuning cell proliferation and cell elongation [10,22,23,24,25], which in the end ensures that the shoot will grow above the soil, whereas the root anchors the plant in the soil [1]. Shoot growth is primarily oriented towards the light (positive phototropic, negative gravitropic) to ensure the most efficient exposure to the sun to harvest energy, which is converted to sugar and powers plant growth [1,2,26]. In contrast, the root grows along the gravity vector (positive gravitropic, negative phototropic) to anchor it in the soil, where it absorbs nutrients and water [2,3]. Auxin biosynthesis correlates with the sugar levels produced depending on light quality and quantity perceived by the leaves and transported to the roots [1,27,28]. The available auxin orchestrates the establishment of root system architecture including, among others, the length of the primary root, the number of lateral roots, and the outgrowth of root hairs [3,10,28,29,30]. Auxin is actively transported towards the root meristem, where it modulates root growth depending on the requirements in a balance between environmental stimuli and available resources, such as carbohydrates, minerals, and water [2,31]. The information about the energy status and available resources of the plant are sensed by the TARGET OF RAPAMYCIN (TOR) complex, which then activates cellular responses upstream of root hair outgrowth [2] (Figure 1).

## 2. Plant Growth Is Dynamic and Orchestrated by Interwoven Signaling Cascades Orchestrated by TOR

Successful plant growth and productivity are highly dependent on the establishment and maintenance of polarity on the tissue, cell, and subcellular levels [13,32,33]. Plants evolved a fine-tuned network of intracellular processes to adapt to environmental changes, leading to fast adaptation responses and manifesting in the structural reorganization of plant architecture to ensure proper nutrient distribution and growth [16,25,34]. Manifold signaling cascades are simultaneously active along the plant body, which implement exogenous signals and information of available resources and the energy status of the shoot and the root [2,25]. TARGET OF RAPAMYCIN (TOR) is a core hub among signaling cascades to balance cellular responses upon hormonal and nutrient signaling, including root meristem activity and root hair outgrowth [2,35,36,37,38].

### 2.1. TOR Complex Acts as a Hub for Signaling Pathways Sensing the Nutrient Status of the Plant

TOR is a Ser/Thr protein kinase, structurally and functionally conserved among all eukaryotes, and acts as a master regulator of transition between cell proliferation and elongation [39,40]. Furthermore, it inhibits autophagy in nutrient-rich conditions [38] (Figure 1). TOR associates with two other proteins in plants, REGULATORY-ASSOCIATED PROTEIN OF TOR (RAPTOR), which presents substrate proteins to TOR, and LETHAL WITH SEC THIRTEEN 8 (LST8), responsible for stabilizing the complex [2,41,42] (Figure 1). TOR signaling networks are crucial to adjusting metabolism rates during plant growth [2,39,43,44,45]. As a crucial part of the glucose signaling network, the TOR complex modulates the activity of transcription factors, such as E2Fα, to enhance root meristem activity [46]. TOR signaling positively regulates the energy-demanding protein translation machinery, but is inhibited when the energy status of the plant drops [11,47]. In elongated starvation, the plant needs to shut down de novo protein synthesis, and furthermore, the TOR inhibitory effect on autophagy is reversed in *Arabidopsis thaliana* [48], as well in the green alga *Chlamydomonas reinhardtii* [49,50,51]. When the available resources do not cover the plant’s energy demand anymore, cell material undergoes recycling [41,52,53]. The drop in energy levels of the plant is sensed by the SNF1-RELATED KINASE 1 (SnRK1) complex, which serves as a plant’s metabolic sensor to keep track of carbohydrate level changes [39,47,54]. The heterotrimeric SnRK1 complex is composed of one catalytic and two regulatory subunits [41] (Figure 1). Three isoforms of the catalytic subunit are known to be encoded in the *Arabidopsis thaliana* genome, but only SnRK1α1/AKIN10/KIN10 is responsible for most of the SnRK1 activity [41,55]. Under nutrient-rich conditions, SnRK1 activity is kept low, but the reduction of available resources leads to the transcriptional upregulation of SnRK1 levels [41,55]. SnRK1 complex activity further results in the activation of autophagy to regain resources [41,54]. Recently, it has been shown that the overexpression of SnRK1α1 leads to the downregulation of the TOR/S6K/RPS6 phosphorylation cascade, while SnRK1α1 suppression causes the opposite effect [47] The TOR/S6K/RPS6 phosphorylation cascade is supposed to be involved in high energy-demanding polysome abundance and protein translation activity as well as stress responses [56,57]. It has been shown that SnRK1α1 physically interacts with RAPTOR and is able to phosphorylate the TOR complex, thereby potentially downregulating its activity [47] (Figure 1). When energy levels rise again, as is the case of elevated photosynthetic activity, rising sugar levels are followed by an enhanced auxin biosynthesis, whereas elevated auxin levels are connected with the activation of TOR by phosphorylation [2,11,46,56] (Figure 1). Besides sugar levels, the availability of amino acids underpins TOR activity, like tryptophan, which is an important precursor for the biosynthesis of auxins [57,58,59,60,61]. TOR signaling and auxin abundance and distribution are tightly interconnected, as auxin levels decrease upon treatment with TOR inhibitors in the root [62]. Among other amino acids, the abundance of cysteine is critical for plant productivity and is also highly dependent on sufficient sulfur uptake [63]. Sulfur depletion is transduced over TOR signaling, resulting in the downregulation of glucose metabolism and the upregulation of autophagy, followed by reduced meristem activity [63]. Sulfate uptake is tightly coupled with carbohydrate and nitrogen metabolism [63], and TOR signaling is thereby a centerpiece of the integration and coordination of light, hormone, and metabolism sensing and signaling upstream of root and shoot architecture adaptation [11].

### 2.2. TOR Activity Depends on ROP2 Action

TOR activity is also regulated by phosphorylation, which, as described above, correlates with auxin abundance in the presence of glucose [46,64]. Enhanced TOR activity is coupled to increased plant growth rate, fitness, and size [11,65]. In the root, shoot-derived glucose and auxin signaling together activate TOR signaling pathways, leading to the upregulation of cell proliferation in the meristem, which further positively correlates with root hair growth [2,66]. Estradiol-inducible *tor-es* mutant and *raptor1b* roots are impaired in glucose-activated root hair elongation [44,46,67]. Furthermore, TOR inhibitor treatments also suggest a link to auxin biosynthesis and signaling in root hair development [11,62]. Auxin activates the TOR complex over RHO-RELATED PROTEIN FROM PLANTS 2 (ROP2) action [45,68] (Figure 1). To date, the interaction between auxin, ROP2, and TOR upstream of TOR signaling on the whole plant level has been well studied [35], but how ROP2 activity and polar auxin transport are precisely regulated during root hair growth initiation depending on TOR signaling must be still determined. In upcoming paragraphs of the review, we will dissect how far the interplay of auxin signaling, TOR activity, and ROP2 mediate root hair outgrowth in more detail [8,69]. Auxin distribution to the root tip and the redistribution towards the root hair zone are, on the other hand, dependent on TOR activity, showing how exogenous and hormonal signals influence each other [2,11].

## 3. Fine-Tuned Root Hair Outgrowth Ensures Plant Growth and Productivity

Roots are very plastic and can adjust their tissue organization and cell appearance during abiotic stress responses [3,70]. The root is divided into individual zones depending on the cell’s maturation status [2]. The meristematic zone continually delivers new cells, and its activity arrests when the environmental conditions are not beneficial for the plant [2]. After leaving the meristem, cells pass through the transition and elongation zone towards the differentiation zone, undergoing maturation and adaptation steps according to the growth conditions of the whole plant [2]. Epidermis cells of the root can be primed while outgrowing the meristem by hormonal signaling events to become root hair cells [9]. Root hair length and density are crucial to anchor the plant in the soil and to enlarge the surface for nutrient and water uptake [2,71]. Both auxin- and TOR-regulated signaling events are dominant regulators of root development and adaptation, mediating tight communications between shoot and root [2,11,72] (Figure 1).

### 3.1. Root Hair Cell Priming, Root Hair Positioning, and Elongation Are Regulated by Auxin

Auxin gradients in the root tip prime root hair cells, the trichoblasts, which grow polar expanding, tubular extensions, the root hairs to enlarge the surface of the root [8,73,74,75,76]. The root epidermis is divided into trichoblasts, root hair cells, and atrichoblasts, non-root hair cells [8]. Cell fate establishment requires fine-tuned cellular events, including the activity of specific receptor-like protein kinases and transcriptional regulators, substantially depending on auxin availability and signaling [75,77,78,79], which are described in Section 4. PIN2 facilitates auxin distribution from the very root tip shootwards, over the lateral root cap and epidermis cells, and back to the root tip over the cortex, resembling a reverse fountain [21,80,81]. In the two cell files of the epidermis, the trichoblasts and atrichoblasts, PIN2 undergoes differential intracellular trafficking, showing higher internalization rates, followed by lower protein abundance at the plasma membrane (PM) in trichoblasts [82]. Auxin transport rates differ among the cell files, and it is suspected that the atrichoblasts act as auxin reservoirs for the trichoblasts [83]. Fine-tuned spatial and temporal auxin distribution triggers transcriptional events responsible for priming root hair cells or orchestrating polar root hair outgrowth in mature cells [9,10] (Figure 1). Mutants of key players of auxin signaling and transport show severe root hair morphology, spacing, and length phenotypes [10,18,84,85]. When shootward auxin transport is impaired, root hairs are shorter, and this is visible in the knockout mutants of the auxin efflux carrier PIN2 as well as for the auxin influx carrier AUXIN1 (AUX1) [10,86]. With further impairment of auxin distribution, like in the triple mutant *aux1 ethylene-insensitive2 (ein2) gnomeb (gneb)*, root hairs appear randomly along the root hair cell, and correct positioning can be restored by auxin application rootward of the trichoblast [10,87]. Auxin signaling depending on TIR1/AFB-Aux/IAA action underpins root hair density (initiation) and length (elongation) [86]. Finally, auxin regulates cell wall properties by initiating its loosening, a crucial event during cell elongation, by activating signaling cascades and transport of structural proteins to the PM, which modify cell wall composition [88,89].

### 3.2. Polar Auxin Transport Is Crucial for On-Point Auxin Distribution

**Figure 2:**
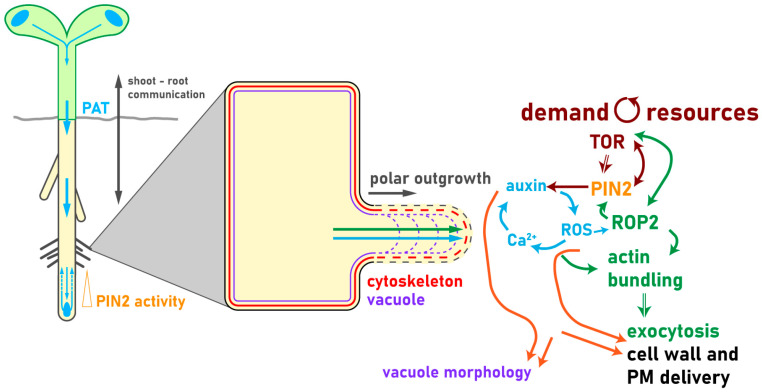
PIN2 orchestrates shootward polar auxin transport as s mediator between exogenous stimuli and root hair outgrowth.

Auxin levels and on-point distribution are modulated by polar auxin transport, biosynthesis, conjugation, perception, and signaling [14,25,90]. Auxin signaling events underpin the reorganization of root hair cells on a subcellular level, including cytoskeleton bundling, vacuolar morphology, cell wall properties, endomembrane trafficking, and membrane composition [25,72,89,91,92,93,94,95]. Polar auxin transport is characterized by active, PM protein-dependent distribution from auxin source to sink, including importers, like the AUXIN1/LIKE-AUX1 (AUX1/LAX) protein family [96,97,98,99] or the nitrate and auxin transporter NITRATE TRANSPORTER 1.1 (NRT1.1) [100,101,102]. AUX1 in the root epidermis is predominantly expressed in non-root hair cells, but its overexpression still enhances root hair outgrowth, while the knockout mutant shows shorter root hairs [9,79,103,104]. Several proteins of the ATP-BINDING CASSETTE SUBFAMILYB-TYPE (ABCB) proteins were found to be involved in auxin efflux and influx [105,106,107,108,109]. ABCB4 and ABCB21 may switch from influx to efflux functions, depending on internal auxin concentrations [110,111,112] and ABCB19 was shown to stabilize PIN1 at the PM [113]. The main occurring auxin in plants is INDOLE-3-ACETIC ACID (IAA), and as a weak acid it requires active export from one cell towards another to maintain long-distance, cell-to-cell transport because it is deprotonated and trapped in the cytoplasm [91,114,115]. Auxin efflux carriers from the PIN family, which are located at the PM, are substantially involved in polarizing auxin flow through the plant body [31,116,117,118,119,120]. Two PIN types can be distinguished by the presence (PIN1, PIN2, PIN3, PIN4, and PIN7) or absence (PIN5 and PIN8) of a long hydrophilic loop in the cytoplasm, whereas PIN6 presents a partially reduced hydrophilic loop [22,81]. PIN1 is involved in the distribution of auxin through the plant and plays a crucial role during embryogenesis [20] and post-embryonic development and growth adaptation [119,121,122,123]. Loss-of-function mutations in PIN1 exhibit a pointed inflorescence stem that fails to produce flowers, emphasizing not only the role of auxin, but also of auxin transport in this process [121,122]. In the root, the redistribution of auxin towards the elongation zone takes place in the root tip and involves the activity of PIN3, PIN4, and PIN7 that localize to the PM of root cap columella cells [81,124,125] Auxin flow continues through PIN2 over the lateral root cap cells and epidermis shootward towards the differentiation zone, and back over the cortex to the root meristem [21,80,81]. Auxin enhances the cell proliferation rate in the root meristem [119,123], and when the energy status of the whole plant drops, auxin distribution from the shoot to the root is reused to stop root growth by downregulating *PIN* expression over the SnRK1 signaling pathway [126]. A recent study showed that glucose-activated TOR phosphorylates and stabilizes PIN2 at the PM, and thereby enhances shootward auxin transport to the root hair zone [127] (Figure 1 and Figure 2).

### 3.3. Auxin Crosstalk with Other Hormones Is Mandatory to Regulate Root Hair Growth

Root hairs allow a rapid expansion of the root surface to elevate the amount of nutrient and water uptake to adjust plant growth and maximize overall plant fitness. The uptake of inorganic phosphate (Pi) is up to 60 times more efficient when root hairs emerge [128]. Therefore, low Pi availability triggers root hair growth [129] and auxin was linked to the orchestration of root hair growth on Pi-deficient media [79]. Furthermore, the integration of environmental stimuli on root hair growth is highly fine-tuned by hormonal crosstalk with other phytohormones, such as ethylene, strigolactones (SLs), and brassinosteroids (BRs) [86,129,130,131,132,133]. BR signaling was shown to stabilize PIN2 at the PM [132,133,134,135]. Cell elongation is modulated by influencing cell wall biosynthesis and cytoskeleton-related functions [136,137]. Auxin and BR activate overlapping sets of genes involved in synergistic interactions in root elongation [138,139]. Crosstalk between auxin and BR signaling changes on a subcellular level the arrangements of actin cytoskeleton bundles [137]. Proper actin bundling is crucial for the elongation ability of root hairs, as *actin2 (act2)* mutants possess shorter root hairs [137], as well as the *pin2* mutant [140]. BR signaling is indispensable to define trichoblast cell fate by regulating the WEREWOLF/GLABRA3/ ENHANCER OF GLABRA3-TRANSPARENT TESTA GLABRA1 transcriptional complex [141]. SL signaling was also linked to cytoskeleton rearrangement [142] and to increased root hair length and density [143]. Cytokinins and light perception of the root were furthermore linked to an important role in root hair growth. cis-Zeatin is needed for root hair elongation and Pi allocation in the root during Pi starvation, which is masked under light, causing stress and interfering with root growth [144]. Most of the published studies regarding root and root hair growth are performed on roots continuously exposed to light, therefore, more detailed studies of root hair emergence and elongation under more natural growth conditions are needed [144,145].

## 4. Cellular Events Downstream of Auxin and TOR Signaling Orchestrate Root Hair Growth

Root hairs serve as a model system for polarity in plant cells [146]. They expand upon the fine-tuned delivery of PM and cell wall components to the root hair tip, driven by cytoskeleton rearrangements and exocytosis [146,147,148]. Auxin gradients and signaling (also dependent on PIN2 action), ROP2 positioning, cytoskeleton rearrangements, the signaling molecules REACTIVE OXYGEN SPECIES (ROS) and Ca^2+^, cell wall remodeling and signaling at PM and cell wall, and, finally, vacuole expansion are well-studied cellular components required for efficient root hair growth and therefore elongation (Figure 2).

Polar auxin transport (PAT) delivers auxin from the shoot to the root depending on the environmental conditions and available resources of the plant (described in Section 3.2). In the root tip, the auxin efflux carrier PIN-FORMED 2 (PIN2) mediates shootward auxin distribution, which is crucial to deliver auxin to the root hair cell. Auxin signaling events underpin subcellular rearrangement that mediates proper root hair tip outgrowth (described in Section 4). Efficient, polar root hair tip elongation primarily depends on the proper establishment of secondary messenger gradients (REACTIVE OXYGEN SPECIES (ROS) and Ca^2+^, described in Section 4.1), subcellular relocalization of ROP2, and rearrangement of the cytoskeleton (described in Section 4.2). Auxin signaling mediates the gene expression of ROS-producing enzymes, *ROP2*, and further key elements, keeping a positive feedback loop at the elongating root hair tip plasma membrane (PM). Cell wall modifications, including loosening by ROS and pH changes and the delivery of new cell wall material by exocytosis, are crucial to allow a steady enlargement of the root tip without bursting. The vacuole stabilizes the expanding root tip from the inside, which is mediated by signaling cascades communicating at the plasma membrane between cell wall and cytoplasm (described in Section 4.3).

### 4.1. Signaling Molecules Initiate a Positive Feedback Loop during Root Hair Growth

ROS regulate root and root hair development downstream of auxin signaling [149,150]. Changed levels of ROS and Ca^2+^ are the primary regulators of root hair emergence and elongation [151,152,153,154], followed immediately by cytoskeleton bundling and rearrangement at the root hair tip [18]. ROS signaling is a fundamental regulatory element of plant growth, including the fine-tuning of tropistic reactions via auxin flow regulation [8,144,155,156]. ROS produced by NICOTINAMIDE ADENINE DINUCLEOTIDE PHOSPHATE-OXIDASE(NOX) is needed to stimulate root hair elongation [150,157,158]. Furthermore, ROS accumulation is crucial for root hair tip outgrowth by modulating cell wall properties [152], regulating the rearrangement and abundance of the secondary messenger, cytoskeleton arrangement, and PM remodeling [150,159,160], and pH changes in the apoplast upon auxin efflux [8]. The calcium gradient at the root hair tip controls the direction of growth, and Ca^2+^ is a universal second messenger, crucial to modulate developmental plasticity in plants [8]. In the emerging root hair tip, it facilitates the fusion of exocytotic vesicles with the apical plasma membrane and subsequent delivery of cell wall cargo to the expanding cell wall [161].

### 4.2. Molecular Key Players of Root Hair Initiation and Elongation

ROPs are GTP-binding proteins acting as molecular switches, and some of them are crucial for root elongation events by regulating cell shape and auxin responses [159,162,163]. ROPs interact with membrane lipids upon posttranslational modifications and thereby transduce external and intracellular stimuli [164]. ROP2 and TOR can interact physically, resulting in TOR phosphorylation, activating TOR signaling pathways [68]. ROP2 is targeted to the PM region of the trichoblast where the root hair will emerge, to polarize subcellular organization to allow root hair outgrowth [165] and is followed by enhanced ROS production at the expanding root hair tip [166,167]. The activation of Ca^2+^ influx followed by increased cytoplasmic Ca^2+^ levels further enhance ROS production, creating a positive feedback loop [168]. Overexpression of ROP2 results in PIN2 accumulation in the root tip, and glucose activation of BR through the actin configuration stabilizes PIN2 at the PM [137,169]. ROP2 is a positive regulator of root hair growth and occurs slightly before the emerging bulge is visible [170] to initiate the positive feedback loop of ROS and Ca^2+^ fluxes responsible for triggering root hair elongation [8,165,166,167]. This triggers changes in the PM composition [171], resulting in the differential bundling of microtubules and actin at the root hair tip [172,173]. ROP2 activates ROS generation through NADPH oxidase action [150,166,174]. Plants overexpressing ROP2 have longer hairs than wild-type plants [69] and multiple root hair outgrowths per trichoblast [175] suggest that its function is needed during all stages of root hair growth. ROP2 is linked to the fine-tuning of filamentous actin (F-actin) bundling [172,176]. The tubular shape and polar growth of root hairs are maintained by correctly assembled microtubules [177] as well as actin bundles [172]. Actin is further essential for bulge site selection and tip growth [18,178,179,180]. F-actin supports cytoplasmic streaming in the cortical cytoplasm, and the distribution of actin bundles results in root hair tip growth inhibition [8,147,181].

### 4.3. Membrane Composition and Endomembrane Trafficking Is Fine-Tuned during Root Hair Growth

Correct membrane composition and fine-tuned endomembrane trafficking are key components of root hair positioning and elongation and are characterized by specific phosphoinositide patterning [182]. Membrane composition is maintained by an interplay of several kinases, like PHOSPATIDYLINOSITOL 4-OH KINASE BETA1 and 2 (PI4Kβ1, PI4Kβ2), which play an essential role in the regulation and delivery of secretory cargo to the tips of growing root hairs [183,184,185]. Furthermore, 1-PHOSPHATIDYLINOSITOL-4-PHOSPHATE 5-KINASE3 (PIP5K3) is required for appropriate tip growth control in root hairs [186,187] by mediating the recruitment of proteins with PI-4P or PI-4,5P2 binding domains, which modulate tip elongation [188,189]. PIP5K3 defines the accumulation site for ROP2, and overexpression of PIP5K3 results in multiple root hair outgrowths from a single trichoblast, whereas the mutants possess shorter root hairs compared to the wild type [69,186]. Vesicles transported to the expanding root hair tip by exocytosis secure polar tip growth by delivering freshly synthesized PM and cell wall components [8,190,191,192]. TOR signaling events modulate cell wall composition by orchestrating galactan/rhamnogalacturonan-I and arabinogalactan protein components over a signaling cascade including REPRESSOR OF LRX1 (ROL5), a mitochondrial protein and major source of ROS, and the extracellular protein LRR-EXTENSIN1 (LRX1) [193]. *Lrx1* mutants develop aberrant root hairs, and TOR signaling can reverse the phenotype by changing the cell wall composition in the same way as it is detected in *rol5* mutants [193]. The maintenance of cell wall integrity (CWI) during controlled root hair tip expansion is crucial to prevent the expanding root hair tip from bursting [89,194]. The PM located kinase FERONIA (FER) modulates the positioning of ROP2, followed by stabilizing actin filaments at the PM [167,195]. FER signaling incorporates cell growth, sensing, and the maintenance of CWI and is linked to ROS activity regulated by ROP2, as *fer* mutants exhibit impaired auxin-induced root hair growth, which can be rescued by overexpressing the FER binding partner ROP2 [167]. Recently, it was also shown how crucial FER is to maintain root hair elongation under elevated temperatures, as the mutant stops root hair tip growth [196]. In the root tip, *fer-4* shows aberrant PIN2 polarity, probably because of diminished F-actin cytoskeleton assembly, which interferes with proper auxin gradient establishment [197]. The expanding root hair tip is almost fully filled with the vacuole, surrounded by a thin layer of highly polarized cytoplasm [8]. Vacuolar morphology has to date been extensively studied during the elongation processes of cells in the primary root tip, where it is crucial to maintain the expansion of the elongating cell without investing excessive resources to build up cytoplasmic content and stability [94,196,197,198,199,200]. It highly depends on intracellular auxin concentration [23,82,201] and signaling pathways partially regulated by FER [202,203]. To date, detailed studies on vacuolar integrity in the expanding root hair tip are lacking.

## 5. Summary and Outlook

Root hair outgrowth is orchestrated upon the appropriate spatial distribution of auxin, which is modulated depending on the overall status of the plant. Energy in the form of sugar and other available nutrients is sensed through the whole plant body and the information is transmitted over the molecular hub TOR complex, which is involved in auxin flow modulation between shoot and roots. In the root tip, auxin is redistributed by the auxin efflux carrier shootwards to the root hairs, where auxin-mediated cellular processes orchestrate polar hair outgrowth through specialized cells. Although manifold studies were published describing every aspect leading to proper root hair outgrowth, some questions are still open. Recent studies showed that direct light illumination interferes with root hair elongation, which is more evident during additive stress responses, therefore, more intense studies on roots grown shaded from light are needed. Furthermore, although much is known about the vacuolar fusion of elongating cells in the elongation zone, further studies are necessary to unravel the mechanisms controlling root hair branching, and the expansion of the vacuole during root hair tip elongation.

## Data Availability

No new data were created or analyzed in this study. Data sharing is not applicable to this article.
